# Laboratory Evaluation of a Vertical Vibration Testing Method for an SMA-13 Mixture

**DOI:** 10.3390/ma13194409

**Published:** 2020-10-03

**Authors:** Yingjun Jiang, Jiangtao Fan, Jinshun Xue, Changqing Deng, Yong Yi, Fuyu Wang

**Affiliations:** 1 Key Laboratory for Special Area Highway Engineering of Ministry of Education, Chang’ an University, Xi’an 710064, China; jyj@chd.edu.cn (Y.J.); changqingdeng@chd.edu.cn (C.D.); 2 School of Civil Engineering and Architecture, Hubei University of Arts and Science, No.296, Longzhong Road, Xiangyang 441053, China; jinshunx@chd.edu.cn; 3 College of Transportation, Jilin University, Changchun 130012, China; 2018121174@chd.edu.cn

**Keywords:** asphalt mixture, vertical vibration testing method, mineral aggregate gradation, physical properties, mechanical properties

## Abstract

In order to simulate the on-site compaction conditions of a Stone Matrix Asphalt (SMA) mixture, The Vertical Vibration Testing Method (VVTM), Superpave Gyratory Compactor (SGC), and Marshall method are used to test the SMA-13 mixture, and the physical and mechanical properties of the asphalt mixture designed by these three methods are tested. Subsequently, the influences of the molding method on the mechanical properties are compared. The influence of vibration compaction time on the volume parameters of the SMA mixture is studied. Following the heavy traffic compaction standards, the vibration compaction time of the SMA mixture is determined. The results show that the densities of the heavy Marshall specimen, VVTM specimen, and SGC specimen are 1.018 times, 1.019 times, and 1.015 times greater than that of the standard Marshall specimen, respectively. The passing rate of the 4.75 mm aggregate of the standard Marshall specimen is 29.9%, and that of the VVTM specimen and SGC specimen is 31.1% and 30.5%, respectively, while that of the heavy Marshall specimen is 34.5%. The mechanical strength of the specimen can be greatly improved as the density increases. On the other hand, by the same compaction work, the mechanical strength of the VVTM specimens can be increased by at least 7% compared with the heavy Marshall specimen. The mechanical strength of the VVTM specimen is increased by at least 22% compared with the standard Marshall specimen. The results also show that under the optimal asphalt-aggregate ratio and the same compaction work, the compressive strength and shear strength of the VVTM specimens are increased by at least 6% and 9%, respectively, compared with the Marshall specimens. In summary, the performance of the asphalt mixture designed by the VVTM is superior, providing a wider choice for future asphalt mixture design.

## 1. Introduction

The Stone Matrix Asphalt (SMA) has excellent road performance, such as rut resistance, crack resistance, skid resistance, and durability; thus it has been widely used worldwide [1]. Owing to the different compaction methods of the SMA mixture, the performance of the aggregate mixture will also vary greatly, resulting in differences in the physical and mechanical properties and road performance. Initially, the SMA mixture was performed by tamping, hand ramming, etc. However, with the development of technology, the Marshall method, the Superpave Gyratory Compactor (SGC) method [2,3,4,5] and the Gyratory Testing Machine (GTM) method are widely used compaction methods in the SMA design at present [6,7,8,9]. The Marshall method was proposed by Bruce Marshall in America in 1939 and was subsequently used all over the world [7]. The Marshall method has been validated owing to its inexpensive equipment and uncomplicated use. In the 1970s, the Marshall method was introduced in China [7], and the number of compaction blows was determined to be 50. With the increase in traffic volume, in the 1980s, the number of compaction blows was adjusted to be 75 and has been used up to now. In the past 20 years, China’s traffic has been developing towards “large flow, heavy axle load, high tire pressure”, and some disadvantages of the Marshall method have become obvious [10,11,12]. The SMA pavements designed by the Marshall method with early ruts, oil spills, and water seepage indicate that the Marshall compaction standard cannot meet current traffic requirements [9,13,14,15]. In addition, the Marshall specimen has a poor correlation with the mechanical properties of the site so that the test indicators of the specimen produced by the Marshall method in the laboratory cannot determine the quality of the field pavement very well [9,13,16]. On the other hand, the compaction work of the SGC method and the GTM method is adjustable; the compaction parameters are basically consistent with the current heavy traffic development, and the preparation of the specimen has a strong correlation with the mechanical properties [17,18]. However, the equipment used in the SGC method and the GTM method is expensive, and the design method is relatively unfamiliar to most Chinese engineers; besides, it has not been well promoted in China. The current research on the vibration testing method for the asphalt mixture is still at the stage of exploration, and most of the previous studies on the vibration testing method of the asphalt mixture are limited to the density, mechanical properties, and compaction characteristics of the compaction method [19,20,21], but do not consider whether the compaction effect of indoor vibration is consistent with that of the site. Besides, there is no global uniform specification for the vibratory compaction method. In order to adapt to the modern traffic conditions, the compaction of the mixture in the laboratory must properly simulate the compaction in real conditions [22,23]. Research results so far indicate that slab-compacted specimens are more closely correlated with cores sampled from the road surface than Marshall specimens [24]. Our research group proposes pavement materials with the Vertical Vibration Testing Method (VVTM) for asphalt mixtures by analyzing the compaction principle of vibratory compaction and deeply studies the maximum compaction capacity of all kinds of existing construction equipment [25,26,27]. Subsequently, the correlation between the VVTM specimen and the physical and mechanical properties of the laboratory-produced samples is evaluated. The results indicate that the correlation between the mechanical strength of specimens produced by the VVTM in the laboratory and the sample from the field is as high as 90% [28,29]. The results also show that VVTM has a good effect particularly on cement stabilized base material and densely graded asphalt mixtures. Moreover, the equipment used to produce specimen is cheaper than the gyratory compactor. Therefore, the vibration compaction method has begun to attract more and more attention in recent years [30]. The research of the vibration compaction method primarily focuses on cement stabilized macadam, densely graded asphalt mixtures, cement cold recycled mixtures, emulsified asphalt cold recycled mixtures, and so on, and have made lots of achievements, but little correlation research related to the SMA mixture by vibration compaction method. Therefore, in order to verify the applicability of the VVTM in the SMA-13 mixture, this paper investigates the effect of different compaction methods (the Marshall method, SGC method, and VVTM) on the physical and mechanical properties of the SMA-13 mixture. The reliability of the VVTM is verified through laboratory tests, and then, we compare the VVTM with the Marshall method and the SGC method. These results can be used as a reference in engineering practice.

## 2. Materials and Methods

### 2.1. Materials

The asphalt used in this test was Styrene-butadiene-styrene (SBS) modified asphalt from South Korea Shuanglong, and its technical properties are listed in Table 1. The coarse aggregate in this test was basalt crushed stone from Shangluo, Shaanxi. The fine aggregate in this test was limestone machine-made sand produced by Shaanxi Hengyutai Construction Engineering Co., Ltd., and the mineral powder was produced by Luonan Zhengtai Mining Co., Ltd., in Shangluo, Shaanxi. All of the materials meet the technical requirements of the Technical Specification for Construction of Highway Asphalt Pavements (JTG F40-2004) [31].

### 2.2. Gradation

The mineral aggregate gradation [32] composition of the SMA-13 [33] mixture is shown in Table 2. In this test, the asphalt-aggregate ratio of the SMA-13 mixture was 5.7%, and the gradation used was the middle of the SMA-13 standard gradation range.

### 2.3. Test Methods

The mechanical evaluation indicators in this study include four aspects, the Marshall Stability (MS), the compressive strength (R_C_), the splitting strength (R_T_), and the shear strength (τ_d_).

#### 2.3.1. Marshall Stability

The MS is the stability index of the asphalt mixture designed by the Marshall method to evaluate the high temperature stability of the asphalt mixture. According to the Standard Test Methods of Bitumen and Bituminous Mixtures for Highway Engineering (JTG E20-2011) [33], a cylindrical specimen with a diameter of 101.6 mm and a height of 63.5 ± 0.13 mm was made, and the temperature was kept at 60 °C. In the installation of the Marshall stability meter, pressure was applied at a loading speed of 50 ± 5 mm/min, and the load when the specimen breaks is the MS value.

#### 2.3.2. Compressive Strength

R_C_ was evaluated by the uniaxial compression test of the asphalt mixture. According to the Standard Test Methods of Bitumen and Bituminous Mixtures for Highway Engineering (JTG E20-2011) [33], a cylindrical specimen with a diameter of 100 mm ± 2 mm and a height of 100 mm ± 2 mm was made, and the temperature was kept at 60 °C. The universal material testing machine loaded uniformly at a loading rate of 2 mm/min until the specimen broke. The load at the time of failure is the R_C_ value.

#### 2.3.3. Splitting Strength

R_T_ is an index to evaluate the low-temperature crack resistance of asphalt mixtures. According to the Standard Test Methods of Bitumen and Bituminous Mixtures for Highway Engineering (JTG E20-2011) [33], a cylindrical specimen with a diameter of 101.6 mm ± 0.25 mm and a height of 63.5 ± 1.3 mm was made, and the temperature was kept at 15 °C. The loading rate used by the testing machine was 50 mm/min. The load and deformation were recorded, and finally, the R_T_ value of the specimen was calculated.

#### 2.3.4. Shear Strength

τ_d_ is an index to evaluate the resistance of the asphalt mixture to shear sliding. The uniaxial penetration test was used to evaluate the τ_d_ of the asphalt mixture. In the test, the loading rate was controlled by the electronic universal testing machine, and then the required data were obtained by the machine. The test parameters are as follows: the test temperature was 60 °C; the loading rate was 1 mm/min; the pressure head diameter was 38 mm [28,29,31,33,34,35].

The shear strength of the asphalt mixture is calculated according to Equations (1) and (2).

(1) $$R_{g}=\frac{4P}{{\pi d}^{2}}$$

(2) $$\tau_{d}=0.339\times R_{g}$$

where R_g_ is the vertical compressive stress of the mixture, P is the maximum load to damage the specimen, τ_d_ is the shear strength of the mixture, and d is the diameter of the penetration rod.

## 3. Vertical Vibration Testing Method

### 3.1. Construction of the Vertical Vibration Testing Equipment

The VVTM is based on the volume design concept. The main differences between the Marshall method and the VVTM are the compaction method and the volume parameter requirements of the specimens. The VVTM is more similar to the on-site construction situation, and the volume parameters of the VVTM are in line with the heavy traffic compaction standards and are particularly suitable for the rapid growth of China’s traffic volume.

In consideration of the operability of indoor instruments, combined with the current internal vibratory vibrator structure of the vibratory roller [16], the Vertical Vibration Testing Equipment (VVTE) was selected to imitate the unique working principles and structural characteristics of the directional vibratory roller. The VVTE mainly includes three parts: control system, vibration system, and power equipment. When the VVTE works, the horizontal components cancel each other, and the vertical components overlap with each other, forming a sinusoidal oscillating force in the vertical direction, which make the VVTE generate vertical vibration and reduce the shear effect of the horizontal force, thus ensuring the stability and the effect of the vertical vibration compaction of the VVTE [28,29].

The adjustable parameters of the VVTE include the static eccentricity, upper system quality, and lower system quality. The parameters were selected to simulate as much as possible the actual compaction effect of the asphalt pavement; the stability and durability of the instrument itself had to be considered. The standard configuration of the VVTE instrument parameters was determined to be in accordance with the related literature [28,29] and is provided in Table 3.

### 3.2. Vibration Time for the Heavy Traffic Compaction Standard

#### 3.2.1. Heavy Traffic Compaction Standard

The final density of the SMA asphalt pavement under modern heavy load traffic is much greater than its design standard density. By using the current technology domestically and abroad, our research group proposes the heavy traffic compaction standard of 1.02 × Marshall density based on the current construction level [28].

#### 3.2.2. Vibration Compaction Time

**Figure 1:**
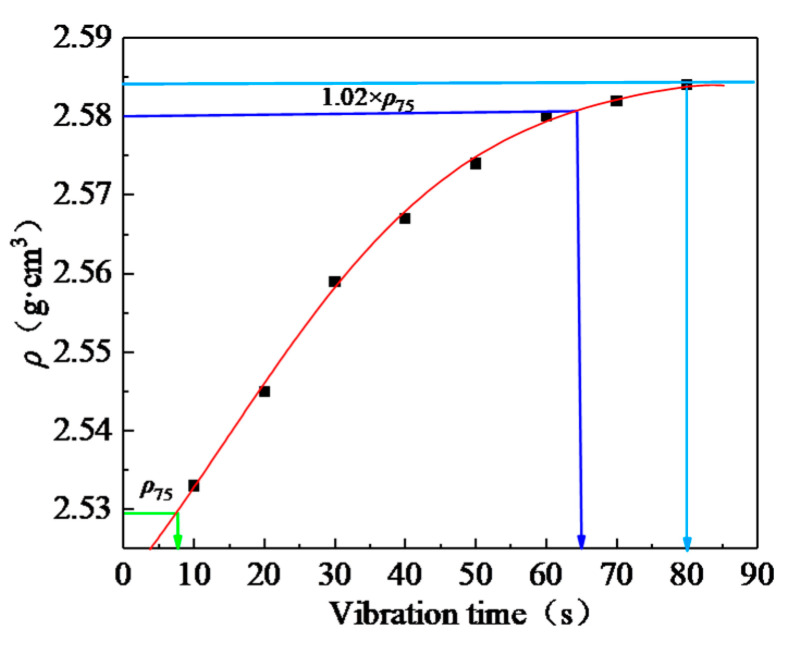
The relationship between the Stone Matrix Asphalt (SMA) density and the vibration time.

The relationship between the SMA mixture density and vibration time is shown in Figure 1.

As can be seen in Figure 1, the density of the SMA mixture specimen increased with the vibration time, and the density did not increase after the vibration time exceeded 80 s. The vibration time corresponding to 1.02 × Marshall density for the heavy traffic compaction standard was about 65 s. Therefore, the vibration time was determined as 65 s in subsequent studies.

### 3.3. Testing Process of the SMA Mixture with VVTM

The preheated test mold and block were taken from the oven, and the SMA mixture that had been well mixed was added into the test mold. Then, a poker was inserted and vibrated evenly along the mold wall 15 times with 10 blows in the middle while adding the materials, and the surface of the mixture after insertion should be arc-shaped and convex. Next, we installed the test specimen (and gasket) of the forming test piece on the VVTE and vibrated the SMA mixture for 65 s. After the compaction was complete, we quickly removed the oil-absorbing paper from the upper and lower surfaces of the specimen and measured the height of the test piece to ensure that the height of the specimen met the specified requirements. After standing for 12 h, a demolding machine was used to remove the specimen.

## 4. Results and Discussion

### 4.1. Analysis of the Physical and Mechanical Properties

**Figure 2:**
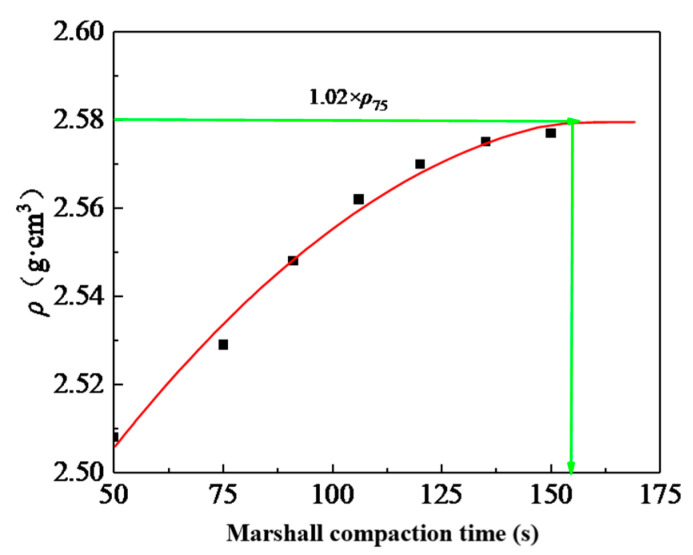
The relationship between the SMA-13 specimen’s density and the Marshall compaction time.

As is well known, for the same raw materials, mineral aggregate gradation, and asphalt-aggregate ratio, the SMA mixture’s properties are affected by the density and aggregate arrangement. The density is related to the compaction work, and the aggregate arrangement is related to the test method. Therefore, it was necessary to evaluate the test method in depth at the same density of the SMA mixture. As shown in Figure 2, the number of double-sided Marshall compactions corresponding to the heavy traffic compaction standard (1.02 × *ρ*75) was 155 (heavy Marshall compaction method).

We analyzed the influence of the standard Marshall method (75 double-sided compactions), heavy Marshall method (155 double-sided compactions), SGC method (100 blows of compaction with rotation), and VVTM (65 s vibration) on the mechanical strength and mineral aggregate grading of the SMA-13 mixture at a 5.7% asphalt-aggregate ratio and in the middle of the SMA-13 standard gradation range, then evaluated the VVTM of the SMA-13 mixture.

#### 4.1.1. Volume Parameter

The volume parameters of the SMA-13 mixture obtained by different methods are provided in Table 4. Volume parameters include volume of air voids (VV), voids in mineral aggregate (VMA), and voids filled with asphalt (VFA).

As presented in Table 4, the density of both the VVTM specimen and the heavy Marshall specimen was close to the density of the heavy traffic compaction standard, which is about 1.02 times the density of the Marshall specimen, and the volume parameters of the two were basically the same, indicating that the compaction power of the two was basically the same. The density of the SGC specimen was slightly smaller than that of the VVTM specimen and the heavy Marshall specimen and was 1.015 times that of the Marshall specimen. This shows that the compaction work of the SGC rotary compaction with 100 blows was slightly less than that of the VVTM and heavy Marshall specimens, but that of the standard Marshall specimen was much greater.

#### 4.1.2. Mineral Aggregate Gradation

The performance of the mineral aggregate gradation had a significant impact on the SMA-13 mixtures. The gradation of the SMA-13 mixture before and after testing by different methods is presented in Table 5.

According to the results presented in Table 5, the following conclusions can be drawn:

(1) After testing by different methods, the SMA-13 minerals were crushed to varying degrees, mainly manifested in an increase in the pass rate of the 4.75 mm coarse aggregate. Zulkuf Kaya et al. [36] found that the use of four different methods to form test pieces causes different degrees of crushing of the aggregate, which is consistent with the conclusions obtained in Table 5.

(2) The 4.75 mm passing rate of the VVTM and SGC specimens was slightly greater than that of the standard Marshall specimen. This is due to the greater compaction work of the VVTM and SGC specimens, but this had little effect.

(3) Although the density of the heavy Marshall specimen was basically the same as that of the VVTM and SGC specimens, the 4.75 mm passing rate of the heavy Marshall specimen was significantly increased, which indicated that the vibration testing method was superior to the Marshall method. This is because Marshall compaction relies on impacts to compact the mixture specimen. During the compaction process, it is relatively difficult for the mineral particles to move. Therefore, the coarse aggregate is easily crushed, showing that both ends of the Marshall specimen are broken. SGC is a rotary compaction method that sets a certain pressure and compaction angle so that the mixture of particles can fully move and rearrange during the compaction process, which can effectively reduce the crushing of the coarse aggregate particles during the compaction process; the VVTM increases the movement of minerals in the SMA mixture by generating vibration pressure waves, especially that of coarse aggregate particles, which reduces the friction between the aggregates, making the SMA mixture denser and the grading of the mineral material less broken.

#### 4.1.3. Mechanical Properties

The mechanical properties and comparison of the SMA-13 mixtures tested by different methods are given in Table 6, where *R*_M_, *R*_ZM_, *R*_S_, and *R*_V_ represent the mechanical strength of the standard Marshall specimen, heavy Marshall specimen, SGC specimen, and VVTM specimen, respectively.

According to the results presented in Table 6, the following conclusions can be drawn:

(1) The impact of compaction work (density): With the same testing method, the heavy Marshall specimen had about a 1.018 times greater density than the standard Marshall specimen, and its mechanical strength increased by at least 14% ((R_ZM_ − R_M_)/R_M_); that is, the mechanical strength of the specimen can be greatly improved as the density increases.

(2) The impact of the testing method: With the same compaction work (density), the mechanical strength of the VVTM specimen was at least 7% greater than that of the heavy Marshall test piece, and the mechanical strength of the VVTM specimen was slightly greater than the SGC specimen, while its density was about 1.004 times greater than that of the SGC specimen. The results showed that the effects of the VVTM and SGC method were basically equivalent, and they were both better than the Marshall method. This is also consistent with the relevant research conclusions of the research group [12,13].

(3) The impact of the compaction work and testing method: Compared with the standard Marshall specimen, the mechanical strength of the VVTM specimen was improved by at least 22%, and the density was approximately 1.019 times greater than that of the standard Marshall specimen, which verified that the testing method and compaction work both had significant impacts on the mechanical properties.

### 4.2. Influence of Molding Mothed on the Mechanical Properties

The raw materials and gradation of the SMA-13 mixture remained unchanged, and the asphalt-aggregate ratio was 4.8%, 5.1%, 5.4%, 5.7%, 6.0%, and 6.3%.

**Figure 3:**
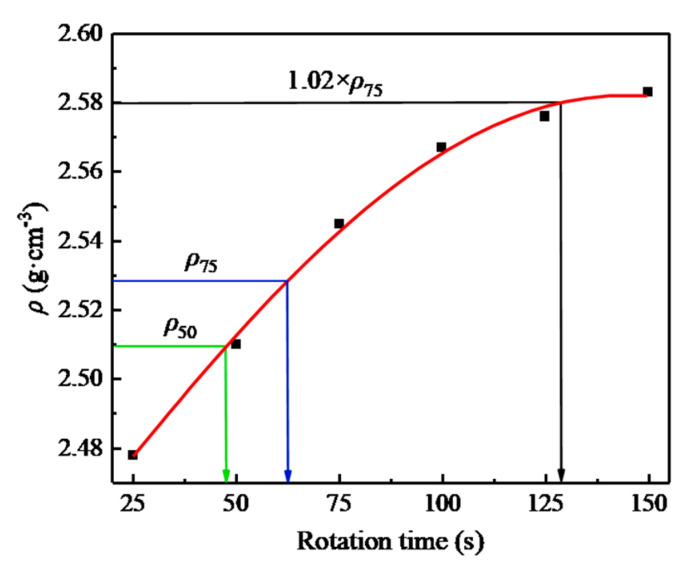
Relationship between the density and rotary compaction times of the SMA-13 specimen.

The molding method adopted the Marshall method, VVTM method, and SGC method; and the compaction work adopted the Marshall double-sided compaction of 50 times (MS50), 75 times (MS75), and 155 times (MS15), vibration testing of 10s (VVTM10) and 65s (VVTM65), and rotary compaction of 50 times (SGC50), 65 times (SGC65), and 130 times (SGC130) (from Figure 3), to study the impact of the compaction methods and compaction work on the asphalt mixture’s performance.

The relationship between the density and compaction times of the SGC specimen is shown in Figure 3. According to the figure, the rotary compaction times corresponding to the density of the Marshall double-sided compaction of 50, 75, and 155 times were 50, 65, and 130, respectively.

#### 4.2.1. Influence of the Compaction Method on the Mechanical Properties

Under the specific conditions of the raw material and mineral aggregate gradation, the mechanical properties of the compacted SMA-13 mixture specimen were a function of the compaction method, compaction work, and asphalt content. Its mechanical parameters included 25 °C R_c_ and 60 °C τ_d_.

**Figure 4:**
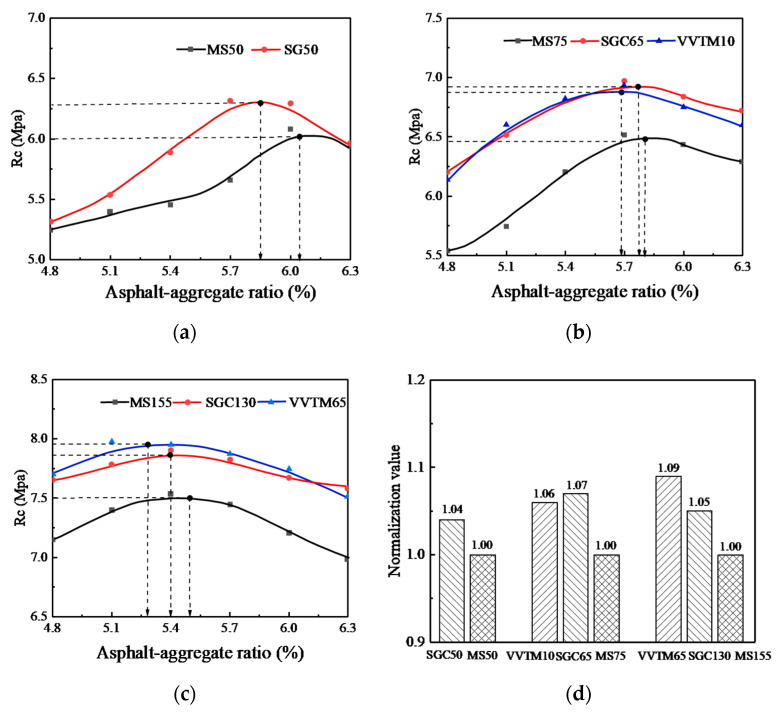
Effects of the compaction method on the compressive strength. (**a**) The corresponding compaction work of the Marshall method of 50 times of double-sided compaction. (**b**) The corresponding compaction work of the Marshall method of 75 times of double-sided compaction. (**c**) The corresponding compaction work of the Marshall method of 155 times of double-sided compaction. (**d**) Comparison of the compressive strength under different compaction work. MS, Marshall Stability.

(1) Compressive strength:

The influence of the compaction methods on the R_c_ of the SMA-13 mixture is shown in Figure 4, in which (a), (b), and (c) are the influence of different compaction methods on the R_c_ and asphalt-aggregate ratio under three kinds of compaction work, and (d) is the value of normalizing the R_c_ peak value of the VVTM and SGC specimens with the Marshall method under each compaction work.

It can be seen from Figure 4 that under the three compaction methods, with the increase of the oil-stone ratio, the R_c_ of the mixture specimens all changed into convex curves, and when the compaction work was the same, the R_c_ of the VVTM and SGC molding specimens was higher than that of the Marshall method.

When the asphalt-aggregate ratio was about 6%, the MS50 and SGC50 specimens showed the peak R_c_. When the asphalt-aggregate ratio was about 5.7%, the VVTM10, SGC65, and MS75 showed the peak R_c_. When the asphalt-aggregate ratio was about 6%, the VVTM65, SGC130, and MS155 showed the peak R_c_. This indicates that the optimal asphalt-aggregate ratio corresponding to the peak R_c_ is a function of the compaction work. The greater the compaction work, the smaller the optimal asphalt-aggregate ratio is.

When the compaction work was the same, the R_c_ of the SGC50 specimen improved by 0.35%~11.5% compared to the MS50 specimen, with an average of 4.5%. At the optimal asphalt-aggregate ratio, it increased by 4%.

The R_c_ of the SGC65 specimen increased by 7~14% compared with the MS75 specimen, with an average of 10%. In the case of the respective optimal asphalt-aggregate ratio, it increased by 7%. The R_c_ of the VVTM10 specimen increased by 5~15% compared to the MS75, with an average of 9%. In the case of the respective optimal asphalt-aggregate ratio, it increased by 6%.

The R_c_ of the SGC130 specimen increased by 5~9% compared with the MS155 specimen, with an average of 6%. In the case of the respective optimal asphalt-aggregate ratio, R_c_ increased by 5%. The R_c_ of the VVTM65 specimen increased by 6~9% compared with the MS155 specimen, with an average of 7%. In the case of the respective optimal asphalt-aggregate ratio, R_c_ increased by 9%.

**Figure 5:**
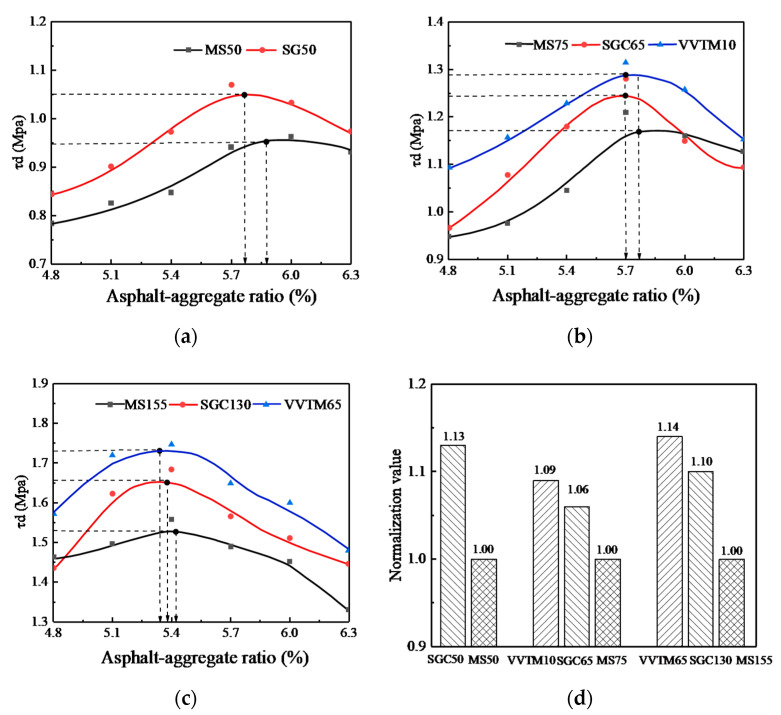
Effects of the compaction method on shear strength. (**a**) The corresponding compaction work of the Marshall method of 50 times of double-sided compaction. (**b**) The corresponding compaction work of the Marshall method of 75 times of double-sided compaction. (**c**) The corresponding compaction work of the Marshall method of 155 times of double-sided compaction. (**d**) Comparison of the shear strength under different compaction work.

(2) Shear strength:

The influence of compaction methods on the τ_d_ of SMA-13 mixture is shown in Figure 5, in which (a), (b) and (c) are the influence of different compaction methods on the τ_d_ and asphalt-aggregate ratio under three kinds of compaction work, and (d) is the value of normalizing the τ_d_ peak value of VTM and SGC specimens with Marshall method under each compaction work.

It can be seen from Figure 5 that no matter which compaction method was adopted, the τ_d_ of the asphalt mixture specimens changed into a convex curve as the asphalt-aggregate ratio increased, and under the same compaction work, the τ_d_ of the VVTM specimen was higher than Marshall specimen and SGC specimen.

When the asphalt-aggregate ratio was about 5.8%, the MS50 and SGC50 specimens showed the peak τ_d_. When the asphalt-aggregate ratio was about 5.7%, the VVTM10, SGC65, and MS75 showed the peak τ_d_. When the asphalt-aggregate ratio was about 5.4%, the VVTM65, SGC130, and MS155 showed the peak τ_d_. This indicates that the optimal asphalt-aggregate ratio corresponding to the peak τ_d_ is a function of the compaction work. The greater the compaction work, the smaller the optimal asphalt-aggregate ratio is.

When the compaction work was the same, the τ_d_ of the SGC50 specimen improved by 5%~15% compared to the MS50 specimen, with an average of 10%. At the optimal asphalt-aggregate ratio, it increased by 13%.

When the asphalt-aggregate ratio was less than 6.0%, the τ_d_ of the SGC65 specimen increased by 2%~13% compared with the MS75 specimen, with an average of 8%. When the asphalt-aggregate ratio was greater than 6.0%, the τ_d_ of the SGC65 specimen was slightly less than the MS75 specimen. In the case of the respective optimal asphalt-aggregate ratio, the τ_d_ of the SGC65 specimen increased by 6% compared to the MS75 specimen. The τ_d_ of the VVTM10 specimen increased by 2%~20% compared to the MS75, with an average of 12%. In the case of the respective optimal asphalt-aggregate ratio, it increased by 9%.

When the asphalt-aggregate ratio was 4.8%, the τ_d_ of the SGC130 specimen was slightly less than the MS155 specimen. When the asphalt-aggregate ratio was greater than 4.8%, the τ_d_ of the SGC130 specimen increased by 4~9% compared with the MS155 specimen, with an average of 7%. In the case of the respective optimal asphalt-aggregate ratio, it increased by 10%. The τ_d_ of the VVTM65 specimen increased by 8~15% compared with the MS155 specimen, with an average of 11%. In the case of the optimal asphalt-aggregate ratio, it increased by 14%.

#### 4.2.2. Influence of Compaction Work on the Mechanical Properties

**Figure 6:**
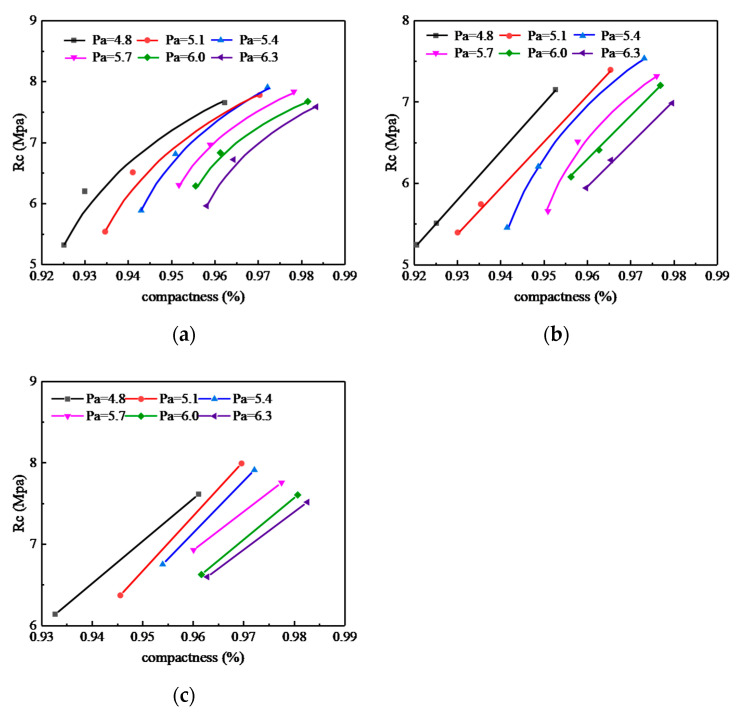
Effects of the compaction degree on the compressive strength under different design methods: (**a**) Marshall method; (**b**) SGC method; (**c**) VVTM.

(1) Compressive strength:

The influence of the compaction work and asphalt-aggregate ratio on the R_c_ of the SMA-13 mixture under different compaction methods is shown in Figure 6.

It can be seen from Figure 6 that under the same compaction method, as the compaction work increased, the R_c_ of the specimen increased linearly.

Compared with the MS50 specimen, under different asphalt-aggregate ratios, the compactness of the MS75 increased by an average of 0.60%; the R_c_ increased by 5~10%, with an average of 9%. The compactness of the MS155 increased by an average of 2.76%; the R_c_ increased by 18~38%, with an average of 30%, that is the compactness increased by 1%. The R_c_ of the Marshall method specimen increased by 13%.

Compared with the SGC50 specimen, under different asphalt-aggregate ratios, the compactness of the SGC65 increased by an average of 0.64%, and R_c_ increased by 9%~18%, with an average of 14%. The compactness of the SGC130 increased by an average of 3.00%, and the R_c_ increased by 22~44%, with an average of 27%, that is the compactness increased by 1%. The R_c_ of the SGC method specimen increased by 15%.

Compared with the VVTM10 specimen, the VVTM65 increased by an average of 2.12%, and the R_c_ increased by 12~26%, with an average of 18%, that is the compactness increased by 1%. The R_c_ of the VVTM specimen increased by 8%.

**Figure 7:**
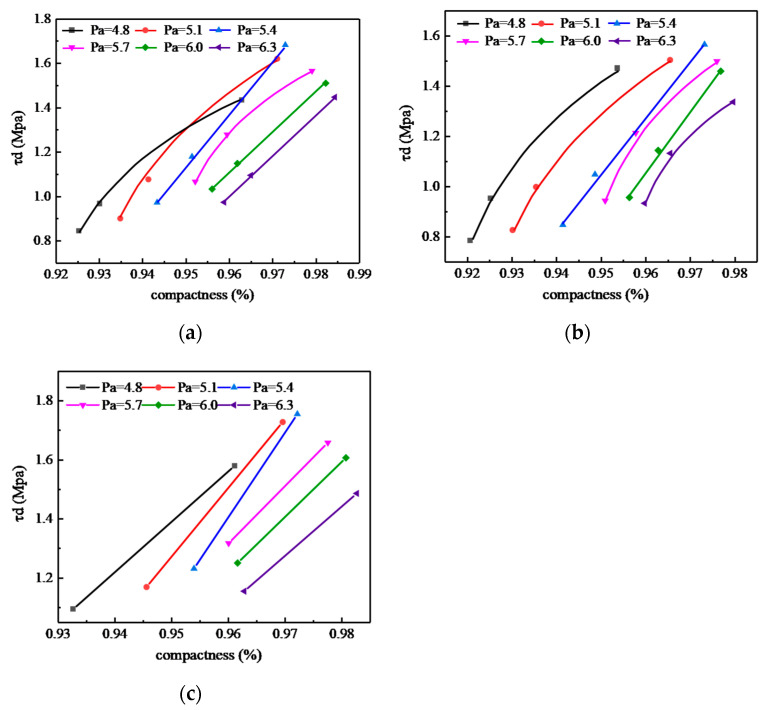
Effects of compaction on the shear strength: (**a**) Marshall method; (**b**) SGC method; (**c**) VVTM.

(2) Shear strength:

The influence of the compaction work and asphalt-aggregate ratio on the τ_d_ of SMA-13 mixture under different compaction methods is shown in Figure 7.

It can be seen from Figure 7 that under the same compaction method, as the compaction work increased, the τ_d_ of the specimen increased linearly.

Compared with the MS50 specimen, under different asphalt-aggregate ratios, the compactness of the MS75 increased by an average of 0.60%; τ_d_ increased by 18~29%, with an average of 22%. The compactness of the MS155 increased by an average of 2.76%; τ_d_ increased by 43~87%, with an average of 68%, that is the compactness increased by 1%. The τ_d_ of the Marshall method specimen increased by 31%.

Compared with the SGC50 specimen, under different asphalt-aggregate ratios, the compactness of the SGC65 increased by an average of 0.64%, and τ_d_ increased by 11~21%, with an average of 17%. The compactness of the SGC130 increased by an average of 3.00%, and τ_d_ increased by 47~80%, with an average of 61%, that is the compactness increased by 1%. The τ_d_ of the SGC method specimen increased by 23%.

Compared with the VVTM10 specimen, the VVTM65 increased by an average of 2.12%, and the τ_d_ increased by 26~47%, with an average of 36%, that is the compactness increased by 1%. The τ_d_ of the VVTM specimen increased by 17%.

## 5. Conclusions

In the research, the relationship between the vibration density and the vibration time of SMA-13 mixture was studied. The physical properties and mechanical properties of the mixture designed by the VVTM, Marshall method, and SGC method were studied and compared. The following conclusions can be drawn:

(1) The VVTM vibration time was 65 s, and the VVTM specimen density was about 1.02 times greater than that of the standard Marshall specimen.

(2) Due to the different compaction principles, different compaction methods had different degrees of crushing effect on the aggregate in the SMA-13 specimen; this is also consistent with Zulkuf Kaya’s research results; the passing rates proved that the crushing effect of the VVTM on the aggregates was less than that of the Marshall method with the same compaction work.

(3) Under the same compaction method, the mechanical strength of the specimen can be greatly improved as the density increases; this point is consistent with the results of the corresponding experiments conducted by the research group on other types of mixtures. The effects of the VVTM and SGC method were basically equivalent, and they both were better than the Marshall method.

(4) The mechanical properties of the asphalt mixtures increased as the asphalt-aggregate ratio increased at the early stage until the peak was reached, then it decreased gradually. Under the optimal asphalt-aggregate ratio and the same compaction work, the R_c_ and τ_d_ of the VVTM specimens were similar to the SGC specimens, while the average R_c_ and τ_d_ of the VVTM specimens increased by at least 4% and 9%, respectively, compared with the Marshall specimens.

(5) When the same method of compaction was used, the mechanical properties of the specimen increased linearly as the compactness increased. The compactness increased by 1%. The R_c_ and τ_d_ of the Marshall specimens increased by 13% and 31%, respectively. Those of the SGC specimens increased by 15% and 23%, respectively. Those of the VVTM specimens increased by 8% and 17%, respectively.

## Figures and Tables

**Table 1:** Technical properties of the SBS modified asphalt.

Indexes	Penetration (25 °C, 100 g, 5 s) (0.1 mm)	Ductility (10 °C) (cm)	Softening Point (°C)	Kinematic Viscosity (135 °C)
Tested Value	71	42.5	82.0	2.0
Standards	60–80	≥30	≥55	≤3.0

**Table 2:** Mineral aggregate gradation.

**Sieve Size (mm)**	16	13.2	9.5	4.75	2.36	1.18	0.6	0.3	0.15	0.075
**Passing Rate (%)**	100.0	95.1	62.6	27.0	20.5	19.0	16.1	13.1	12.1	10.2

**Table 3:** The Vertical Vibration Testing Equipment (VVTE) instrument’s standard parameter configuration.

Working Frequency (Hz)	Nominal Amplitude (mm)	Working Weight (kg)		
		Upper System	Lower System	Gross Weight
37	1.2	108	167	275

**Table 4:** Specimen volume parameters obtained by different testing methods. VVTM, Vertical Vibration Testing Method; SGC, Superpave Gyratory Compactor.

Test Type	Density (g/cm^3^)		VV (%)		VMA (%)		VFA (%)	
	Tested Value	Relative Value	Tested Value	Relative Value	Tested Value	Relative Value	Tested Value	Relative Value
Marshall	2.529	1.000	4.1	1.00	17.1	1.00	75.9	1.00
Heavy Marshall	2.575	1.018	2.4	0.58	15.6	0.91	84.6	1.11
VVTM	2.578	1.019	2.3	0.56	15.5	0.91	85.4	1.13
SGC	2.557	1.015	3.0	0.73	16.1	0.94	81.1	1.07

**Table 5:** Specimen mineral aggregate gradation obtained by different testing methods.

Test Type	Passing Rate (%)									
	16	13.2	9.5	4.75	2.36	1.18	0.6	0.3	0.15	0.075
Marshall	100.0	95.8	64.7	29.9	22.2	20.3	17.2	14.0	13.0	10.9
Heavy Marshall	100.0	96.9	66.9	34.5	23.4	21.1	17.7	14.2	13.1	10.9
VVTM	100.0	96.3	65.6	31.1	22.6	20.6	17.3	14.2	13.1	11.0
SGC	100.0	96.0	65.4	30.5	22.0	20.2	17.0	13.9	12.8	10.7

**Table 6:** The mechanical strength and comparison of the SMA-13 specimen tested by different methods.

Mechanical Strength	*R*_M_	*R*_ZM_	*R*_S_	*R*_V_	*R*_V_/*R*_M_	*R*_V_/*R*_ZM_	*R*_V_/*R*_S_
60 °C Marshall stability (kN)	14.17	17.52	18.49	19.41	1.37	1.11	1.05
20 °C compressive strength (MPa)	6.39	7.32	7.61	7.75	1.21	1.06	1.02
−10 °C splitting strength (MPa)	3.37	4.04	4.23	4.43	1.31	1.10	1.05
60 °C shear strength (MPa)	1.19	1.49	1.52	1.65	1.39	1.11	1.09
